# Modeling the Dynamics of Human Brain Activity with Recurrent Neural Networks

**DOI:** 10.3389/fncom.2017.00007

**Published:** 2017-02-09

**Authors:** Umut Güçlü, Marcel A. J. van Gerven

**Affiliations:** Donders Institute for Brain, Cognition and Behaviour, Radboud UniversityNijmegen, Netherlands

**Keywords:** encoding, fMRI, RNN, LSTM, GRU

## Abstract

Encoding models are used for predicting brain activity in response to sensory stimuli with the objective of elucidating how sensory information is represented in the brain. Encoding models typically comprise a nonlinear transformation of stimuli to features (feature model) and a linear convolution of features to responses (response model). While there has been extensive work on developing better feature models, the work on developing better response models has been rather limited. Here, we investigate the extent to which recurrent neural network models can use their internal memories for nonlinear processing of arbitrary feature sequences to predict feature-evoked response sequences as measured by functional magnetic resonance imaging. We show that the proposed recurrent neural network models can significantly outperform established response models by accurately estimating long-term dependencies that drive hemodynamic responses. The results open a new window into modeling the dynamics of brain activity in response to sensory stimuli.

## 1. Introduction

Encoding models (Naselaris et al., 2011) are used for predicting brain activity in response to naturalistic stimuli (Felsen and Dan, 2005) with the objective of understanding how sensory information is represented in the brain. Encoding models typically comprise two main components. The first component is a feature model that nonlinearly transforms stimuli to features (i.e., the independent variables used in fMRI time series analyses). The second component is a response model that linearly transforms features to responses. While encoding models have been successfully used to characterize the relationship between stimuli in different modalities and responses in different brain regions, their performance usually falls short of the expected performance of the true encoding model given the noise in the analyzed data (noise ceiling). This means that there usually is unexplained variance in the analyzed data that can be explained solely by improving the encoding models.

One way to reach the noise ceiling is the development of better feature models. Recently, there has been extensive work in this direction. One example is the use of convolutional neural network representations of natural images or natural movies to explain low-, mid- and high-level representations in different brain regions along the ventral (Agrawal et al., 2014; Cadieu et al., 2014; Khaligh-Razavi and Kriegeskorte, 2014; Yamins et al., 2014; Güçlü and van Gerven, 2015a; Cichy et al., 2016) and dorsal streams (Güçlü and van Gerven, 2015b; Eickenberg et al., 2016) of the human visual system. Another example is the use of manually constructed or statistically estimated representations of words and phrases to explain the semantic representations in different brain regions (Mitchell et al., 2008; Huth et al., 2012; Murphy et al., 2012; Fyshe et al., 2013; Güçlü and van Gerven, 2015c; Nishida et al., 2015).

Another way to reach the noise ceiling is the development of better response models. There is a long history of estimating hemodynamic response functions (HRFs) in fMRI time series modeling. The standard general linear (convolution) model used in procedures like statistical parametric mapping (SPM) expands the HRF in terms of orthogonal kernels or temporal basis functions that have been motivated in terms of Volterra expansions. Indeed, commonly used software packages such as the SPM software have (hidden) facilities to model second-order Volterra kernels that enable modeling of non-linear hemodynamic effects such as saturation. In reality, the transformation from stimulus features to observed responses is exceedingly complex because of various temporal dependencies that are caused by neurovascular coupling (Logothetis and Wandell, 2004; Norris, 2006) and other more elusive cognitive or neural factors.

Here, our objective is to develop a model that can be trained end to end, captures temporal dependencies and processes arbitrary input sequences for time-continuous fMRI experiments such as watching movies, listening to music or playing video games. Such time-continuous designs are characterized by the absence of discrete experimental events as those found in their block or event-related counterparts. To this end, we use recurrent neural networks (RNNs) as response models in the encoding framework. Recently, RNNs in general and two RNN variants—long short-term memory (Hochreiter and Schmidhuber, 1997) and gated recurrent units (Cho et al., 2014)—in particular have been shown to be extremely successful in various tasks that involve processing of arbitrary input sequences such as handwriting recognition (Graves et al., 2009; Graves, 2013), language modeling (Sutskever et al., 2011; Graves, 2013), machine translation (Cho et al., 2014) and speech recognition (Sak et al., 2014). These models use their internal memories to capture the temporal dependencies that are informative about solving the task at hand. That is, these models base their predictions not only to the information available at a given time, but also to the information that was available in the past. They accomplish this by maintaining an explicit or implicit representation of the past input sequences and use it to make their predictions at each time point. If these models can be used as response models in the encoding framework, it will open a new window into modeling brain activity in response to sensory stimuli since the brain activity is modulated by long temporal dependencies.

While the use of RNNs in the encoding framework has been proposed a number of times (Güçlü and van Gerven, 2015a,b; Kriegeskorte, 2015; Yamins and DiCarlo, 2016a,b), these proposals mainly focused on using RNNs as feature models. In contrast, we have framed our approach in terms of response models used in characterizing distributed or multivariate responses to stimuli in the encoding framework. The key thing that we bring to the table is a generic and potentially useful response model that transforms features to observed (hemodynamic) responses. From the perspective of conventional analyses of functional magnetic resonance imaging (fMRI) time series, this response model corresponds to the convolution model used to map stimulus features (e.g., the presence of biological motion) to fMRI responses. In other words, the stimulus features correspond to conventional stimulus functions that enter standard convolution models of fMRI time series (e.g., the GLM used in statistical parametric mapping).

In brief, we know that the transformation from neuronal responses to fMRI signals is mediated by neuronal and hemodynamic factors that can always be expressed in terms of a non-linear convolution. A general form for these convolutions has been previously considered in the form of Volterra kernels or functional Taylor expansions (Friston et al., 2000). Crucially, it is also well known that RNNs are universal non-linear approximators that can reproduce any Volterra expansion (Wray and Green, 1994). This means that we can use RNNs as an inclusive and flexible way to parameterize the convolution of stimulus features generating hemodynamic responses. Furthermore, we can use RNNs to model not just response of a single voxel but distributed responses over multiple voxels. Having established the parametric form of this convolution, the statistical evidence or significance of each regionally specific convolution can then be assessed using standard (cross-validation) machine learning techniques by comparing the accuracy of the convolution when applied to test data after optimization with training data.

We test our approach by comparing how well a family of RNN models and a family of ridge regression models can predict blood-oxygen-level dependent (BOLD) hemodynamic responses to high-level and low-level features of natural movies using cross-validation. We show that the proposed recurrent neural network models can significantly outperform the standard ridge regression models and accurately estimate hemodynamic response functions by capturing temporal dependencies in the data.

## 2. Materials and methods

### 2.1. Data set

We analyzed the vim-2 data set (Nishimoto et al., 2014), which was originally published by Nishimoto et al. (2011). The experimental procedures are identical to those in Nishimoto et al. (2011). Briefly, the data set has twelve 600 s blocks of stimulus and response sequences in a training set and nine 60 s blocks of stimulus and response sequences in a test set. The stimulus sequences are videos (512 px × 512 px or 20° × 20°, 15 FPS) that were drawn from various sources. The response sequences are BOLD responses (voxel size = 2 × 2 × 2.5 mm^3^, TR = 1 s) that were acquired from the occipital cortices of three subjects (S1, S2, and S3). The stimulus sequences in the test set were repeated ten times. The corresponding response sequences were averaged over the repetitions. The response sequences have already been preprocessed as described in Nishimoto et al. (2011). Briefly, they have been realigned to compensate for motion, detrended to compensate for drift and z-scored. Additionally, the first six seconds of the blocks were discarded. No further preprocessing was performed. Regions of interests were localized using the multifocal retinotopic mapping technique on retinotopic mapping data that were acquired in separate sessions (Hansen et al., 2004). As a result, the voxels were grouped into 16 areas. However, not all areas were identified in all subjects (Table 1). The last 45 seconds of the blocks in the training set were used as the validation set.

**Table 1:** **Number of voxels per subject and area**.

	**V2**	**V3**	**V1**	**IPS**	**V4**	**LOC**	**V7**	**MT+**	**V3A**	**V3B**	**VO**	**EBA**	**OFA**	**RSC**	**pSTS**	**TOS**
S1	1,477	1,141	994	2,251	734	885	0	466	252	256	410	0	0	71	45	0
S2	1,659	1,360	1,043	0	1032	614	400	174	337	223	267	319	246	128	0	0
S3	1,377	1,131	1,366	893	750	408	583	263	282	225	0	131	91	8	16	41

### 2.2. Problem statement

Let **x**^*t*^ ∈ ℝ^*n*^ and **y**^*t*^ ∈ ℝ^*m*^ be a stimulus and a response at temporal interval [*t, t* + 1], where *n* is the number of stimulus dimensions and *m* is the number of voxel responses. We are interested in predicting the most likely response **y**^*t*^ given the stimulus history **X**^*t*^ = (**x**^0^,…,**x**^*t*^):

(1) $$\begin{array}{rcl} {\hat{\text{y}}}^{t} & = & \arg\underset{{\text{y}}^{t}}{\max}\mathrm{Pr}\left({\text{y}}^{t}|{\text{X}}^{t}\right) \end{array}$$

(2) $$\begin{array}{ll} = & \text{g}\left(\phi \left({\text{x}}^{0}\right),\ldots ,\phi \left({\text{x}}^{t}\right)\right) \end{array}$$

where Pr is an encoding distribution, ϕ is a feature model such that ϕ (·) ∈ ℝ^*p*^, *p* is the number of feature dimensions, and **g** is a response model such that **g** (·) ∈ ℝ^*m*^.

**Figure 1:**
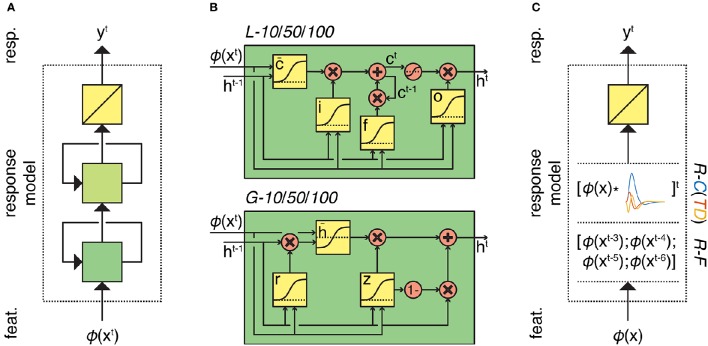
**Overview of the response models**. **(A)** Response models in the RNN family. All RNN models process feature sequences via two (recurrent) nonlinear layers and one (nonrecurrent) linear layer but differ in the type and number of artificial neurons. *L-10/50/10* models have 10, 50, or 100 long short-term memory units in both of their hidden layers, respectively. Similarly, *G-10/50/10* models have 10, 50, or 100 gated recurrent units in both of their hidden layers, respectively. **(B)** First-layer long short-term memory and gated recurrent units. Squares indicate linear combination and nonlinearity. Circles indicate elementwise operations. Gates in the units control the information flow between the time points. **(C)** Response models in the ridge regression family. All ridge regression models process feature sequences via one (nonrecurrent) linear layer but differ in how they account for the hemodynamic delay. *R-C(TD)* models convolve the feature sequence with the canonical hemodynamic response function (and its time and dispersion derivatives). *R-F* model lags the feature sequence for 3, 4, 5, and 6 s and concatenates the lagged sequences.

In order to solve this problem, we must define the feature model that transforms stimuli to features and the response model that transforms features to responses. We used two alternative feature models; a scene description model that codes for low-level visual features (Oliva and Torralba, 2001) and a word embedding model that codes for high-level semantic content. We used two response model families that differ in architecture (recurrent neural network family and feedforward ridge regression family) (Figure 1). In contrast to standard convolution models for fMRI time series, we are dealing with potentially very large feature spaces. This means that in the absence of constraints the optimization of model parameters can be ill posed. Therefore, we use dropout and early stopping for the recurrent models, and *L*^2^ regularization for the feedforward models.

### 2.3. Feature models

#### 2.3.1. High-level semantic model

As a high-level semantic model we used the word2vec (W2V) model by Mikolov et al. (2013a,b,c). This is a one-layer feedforward neural network that is trained for predicting either target words/phrases from source-context words (continuous bag-of-words) or source context-words from target words/phrases (skip-gram). Once trained, its hidden states are used as continuous distributed representations of words/phrases. These representations capture many semantic regularities. We used the pretrained (skip-gram) W2V model to avoid training from scratch (https://code.google.com/archive/p/word2vec/). It was trained on 100 billion-word Google News dataset. It contains 300-dimensional continuous distributed representations of three million words/phrases.

We used the W2V model for transforming a stimulus sequence to a feature sequence on a second-by-second basis as follows: First, each one second of the stimulus sequence is assigned 20 categories (words/phrases). We used the *Clarifai* service (http://www.clarifai.com/) to automatically assign the categories rather than annotating them by hand. *Clarifai* provides a web-based video recognition application, which internally uses a pretrained deep neural network to automatically tag the contents of the video frames on a second-by-second basis. Then, each category is transformed into continuous distributed representations of words/phrases. Next, these representations are averaged over the categories. This resulted in a 300-dimensional feature vector per second of stimulus sequence (*p* = 300).

#### 2.3.2. Low-level visual feature model

As a low-level visual feature model we used the GIST model (Oliva and Torralba, 2001). The GIST model transforms scenes into spatial envelope representations. These representations capture many perceptual dimensions that represent the dominant spatial structure of a scene and have been used to study neural representations in a number of earlier work (Groen et al., 2013; Leeds et al., 2013; Cichy et al., 2016). We used the implementation that is provided at: http://people.csail.mit.edu/torralba/code/spatialenvelope/.

We used the GIST model for transforming a stimulus sequence to a feature sequence on a second-by-second basis as follows: First, each 16 non-overlapping 8 × 8 regions of all 15 128 × 128 frames in one second of the stimulus sequence are filtered with 32 Gabor filters that have eight orientations and four scales. Then, their energies are averaged over the frames. This resulted in a 512-dimensional feature vector per second of stimulus sequence (*p* = 512).

### 2.4. Response models

#### 2.4.1. Ridge regression family

The response models in the ridge regression family predict feature-evoked responses as a linear combination of features. Each member of this family differs in how it accounts for the hemodynamic delay.

The *R-C* model (i) convolves the features with the canonical hemodynamic response function (Friston et al., 1994) and (ii) predicts the responses as a linear combination of these features:

(3) $$\begin{array}{rcl} {\hat{\text{y}}}^{t} & = & {\left({\text{H}}_{c}{\text{F}}_{c}{\text{B}}^{⊤}\right)}^{t} \end{array}$$

where ${\text{H}}_{c}\in {\mathbb{R}}^{t\times t}$ is the Toeplitz matrix of the canonical HRF. That is, it is a diagonal-constant matrix that contains the shifted versions of the HRF in its columns. Multiplying it with a signal corresponds to convolution of the HRF with the signal. Furthermore, ${\text{F}}_{c}={\left[\phi \left({\text{x}}^{0}\right),\ldots ,\phi \left({\text{x}}^{t}\right)\right]}^{⊤}\in {\mathbb{R}}^{t\times p}$ and **B** ∈ ℝ^*m*×*p*^ is the matrix of regression coefficients.

The *R-CTD* model (i) convolves the features with the canonical hemodynamic response function, its temporal derivative and its dispersion derivative (Friston et al., 1998), (ii) concatenates these features and (iii) predicts the responses as a linear combination of these features:

(4) $$\begin{array}{rcl} {\hat{\text{y}}}^{t} & = & {\left(\left[{\text{H}}_{c}{\text{F}}_{c},{\text{H}}_{ct}{\text{F}}_{c},{\text{H}}_{cd}{\text{F}}_{c}\right]{\text{B}}^{⊤}\right)}^{t} \end{array}$$

where ${\text{H}}_{ct}\in {\mathbb{R}}^{t\times t}$ is the Toeplitz matrix of the the temporal derivative of the canonical HRF, ${\text{H}}_{cd}\in {\mathbb{R}}^{t\times t}$ is the Toeplitz matrix of the the dispersion derivative of the canonical HRF and **B** ∈ ℝ^*m*×3*p*^ is the matrix of regression coefficients.

The *R-F* model is a finite impulse response (FIR) model that (i) lags the features for 3, 4, 5, and 6 s (Nishimoto et al., 2011), (ii) concatenates these features and (iii) predicts the responses as a linear combination of these features:

(5) $$\begin{array}{rcl} {\hat{\text{y}}}^{t} & = & {\text{F}}_{f}{\text{B}}^{⊤} \end{array}$$

where ${\text{F}}_{f}={\left[\phi \left({\text{x}}^{t-3}\right),\phi \left({\text{x}}^{t-4}\right),\phi \left({\text{x}}^{t-5}\right),\phi \left({\text{x}}^{t-6}\right)\right]}^{⊤}\in {\mathbb{R}}^{t\times 4p}$ and **B** ∈ ℝ^*m*×4*p*^ is the matrix of regression coefficients.

We used the validation set for model selection (a regularization parameter per voxel) and the training set for model estimation (a row of **B** per voxel). Regularization parameters were selected as explained in Güçlü and van Gerven (2014). The rows of **B** were estimated by analytically minimizing the *L*^2^-penalized least squares loss function. In related Bayesian models, this corresponds to applying shrinkage priors to the parameters (weights) of our model.

#### 2.4.2. Recurrent neural network family

The response models in the RNN family are two-layer recurrent neural network models. They use their internal memories for nonlinearly processing arbitrary feature sequences and predicting feature-evoked responses as a linear combination of their second-layer hidden states:

(6) $$\begin{array}{r} {\hat{\text{y}}}^{t}={\text{h}}_{2}^{t}{\text{W}}^{⊤} \end{array}$$

where ${\text{h}}_{2}^{t}$ represents the hidden states in the second layer, and **W** are the weights. The RNN models differ in the type and number of artificial neurons.

The *L-10, L-50*, and *L-100* models are two-layer recurrent neural networks that have 10, 50, and 100 long short-term memory (LSTM) units (Hochreiter and Schmidhuber, 1997) in their hidden layers, respectively. Each LSTM unit has a cell state that acts as its internal memory by storing information from previous time points. The contents of the cell state are modulated by the gates of the unit and in turn modulate its outputs. As a result, the output of the unit is not only controlled by the present stimulus alone, but also by the stimulus history. The gates are implemented as multiplicative sigmoid functions of the inputs of the unit at the current time point and the outputs of the unit at the previous time point. That is, the gates produce values between zero and one, which are multiplied by (a function of) the cell state to determine the amount of information to store, forget or retrieve at each time point. The first-layer hidden states of an LSTM unit are defined as follows:

(7) $$\begin{array}{rcl} {\text{h}}^{t} & = & {\text{o}}^{t}⊙\tanh\left({\text{c}}^{t}\right) \end{array}$$

(8) $$\begin{array}{rcl} {\text{o}}^{t} & = & \sigma \left({\text{U}}_{o}{\text{h}}^{t-1}+{\text{W}}_{o}\phi \left({\text{x}}^{t}\right)+{\text{b}}_{o}\right) \end{array}$$

where ⊙ denotes elementwise multiplication, **c**^*t*^ is the cell state, and **o**^*t*^ are the output gate activities. The cell state maintains information about the previous time points. The output gate controls what information will be retrieved from the cell state. The cell state of an LSTM unit is defined as:

(9) $$\begin{array}{rcl} {\text{c}}^{t} & = & {\text{f}}^{t}⊙{\text{c}}^{t-1}+{\text{i}}^{t}⊙{\bar{\text{c}}}^{t} \end{array}$$

(10) $$\begin{array}{rcl} {\text{f}}^{t} & = & \sigma \left({\text{U}}_{f}{\text{h}}^{t-1}+{\text{W}}_{f}\phi \left({\text{x}}^{t}\right)+{\text{b}}_{f}\right) \end{array}$$

(11) $$\begin{array}{rcl} {\text{i}}^{t} & = & \sigma \left({\text{U}}_{i}{\text{h}}^{t-1}+{\text{W}}_{i}\phi \left({\text{x}}^{t}\right)+{\text{b}}_{i}\right) \end{array}$$

(12) $$\begin{array}{rcl} {\bar{\text{c}}}^{t} & = & \sigma \left({\text{U}}_{c}{\text{h}}^{t-1}+{\text{W}}_{c}\phi \left({\text{x}}^{t}\right)+{\text{b}}_{c}\right) \end{array}$$

where **f**^*t*^ are the forget gate activities, **i**^*t*^ are the input gate activities, and ${\bar{\text{c}}}^{t}$ is an auxiliary variable. Forget gates control what old information will be discarded from the cell states. Input gates control what new information will be stored in the cell states. Furthermore, **U**s and **W**s are the weights and **b**s are the biases that determine the behavior of the gates (i.e., the learnable parameters of the model).

The *G-10, G-50*, and *G-100* models are two-layer recurrent neural networks that have 10, 50, and 100 gated recurrent units (GRU) (Cho et al., 2014) in the their hidden layers, respectively. The GRU units are simpler alternatives to the LSTM units. They combine hidden states with cell states and input gates with forget gates. The first-layer hidden states of a GRU unit is defined as follows:

(13) $$\begin{array}{rcl} {\text{h}}^{t} & = & \left(1-{\text{z}}^{t}\right)⊙{\text{h}}^{t-1}+{\text{z}}^{t}⊙{\bar{\text{h}}}^{t} \end{array}$$

(14) $$\begin{array}{rcl} {\text{z}}^{t} & = & \sigma \left({\text{U}}_{z}{\text{h}}^{t-1}+{\text{W}}_{z}\phi \left({\text{x}}^{t}\right)+{\text{b}}_{z}\right) \end{array}$$

(15) $$\begin{array}{rcl} {\text{r}}^{t} & = & \sigma \left({\text{U}}_{r}{\text{h}}^{t-1}+{\text{W}}_{r}\phi \left({\text{x}}^{t}\right)+{\text{b}}_{r}\right) \end{array}$$

(16) $$\begin{array}{rcl} {\bar{\text{h}}}^{t} & = & \tanh\left({\text{U}}_{h}\left({\text{r}}_{t}⊙{\text{h}}^{t-1}\right)+{\text{W}}_{h}\phi \left({\text{x}}^{t}\right)+{\text{b}}_{h}\right) \end{array}$$

where **z**^*t*^ are update gate activities, **r**^*t*^ are reset gate activities and ${\bar{\text{h}}}^{t}$ is an auxiliary variable. Like the gates in LSTM units, those in GRU units control the information flow between the time points. As before, **U**s and **W**s are the weights and **b**s are the biases that determine the behavior of the gates (i.e., the learnable parameters of the model).

The second-layer hidden states are defined similarly to the first-layer hidden states except for replacing the input features with the first-layer hidden states. For each previously identified brain area of each subject, a separate model was trained. That is, the voxels in a given brain area of a given subject shared the same recurrent layers but had different weights for linearly transforming the hidden states of the second recurrent layer to the response predictions. We used truncated backpropagation through time in conjunction with the optimization method Adam (Kingma and Ba, 2014) to train the models on the training set by iteratively minimizing the mean squared error loss function. Dropout (Hinton et al., 2012) was used to regularize the hidden layers. The epoch in which the validation performance was the highest was taken as the best model. The *Chainer* framework (http://chainer.org/) was used to implement the models.

### 2.5. HRF estimation

Voxel-specific HRFs were estimated by stimulating the RNN model with an impulse. Let **x**^−*t*^, …, **x**^0^, …, **x**^*t*^ be an impulse such that **x** is a vector of zeros at times other than time 0 and a vector of ones at time 0. The period of the impulse before time 0 is used to stabilize the baseline of the impulse response. First, the response of the model to the impulse is simulated:

(17) $$\begin{array}{rcl} {\left[{\text{H}}_{r}^{*}\right]}_{-t}^{t} & = & {\text{g}}_{r}\left({\text{x}}^{-t},\ldots ,{\text{x}}^{0},\ldots ,{\text{x}}^{t}\right) \end{array}$$

where ${\left[{\text{H}}_{r}^{*}\right]}_{-t}^{t}=\left({\text{H}}_{r}^{*-t},\ldots ,{\text{H}}_{r}^{*0},\ldots ,{\text{H}}_{r}^{*t}\right)$. Then, the baseline of the impulse response before time 0 is subtracted from itself:

(18) $$\begin{array}{rcl} {\left[{\text{H}}_{r}^{*}\right]}_{-t}^{t} & = & {\left[{\text{H}}_{r}^{*}\right]}_{-t}^{t}-{\text{H}}_{r}^{*-1}. \end{array}$$

Next, the impulse response is divided by its maximum:

(19) $$\begin{array}{ccc} {\left[H_{r}^{*}\right]}_{-t}^{t} & = & {\left[H_{r}^{*}\right]}_{-t}^{t}/\max{\left[H_{r}^{*}\right]}_{-t}^{t}. \end{array}$$

Finally, the period of the impulse response before time 0 is discarded, and the remaining period of the impulse response is taken as the HRF of the voxels:

(20) $$\begin{array}{rcl} {\left[{\text{H}}_{r}\right]}_{0}^{t} & = & {\left[{\text{H}}_{r}^{*}\right]}_{0}^{t}. \end{array}$$

The time when the HRF is at its maximum was taken as the delay of the response, and the time after the delay of the response when the HRF was at its minimum was taken as the delay of undershoot.

### 2.6. Performance assessment

The performance of a model for a voxel was defined as the cross-validated Pearson's product-moment correlation coefficient between the observed and predicted responses of the voxel (*r*)^1^. Its performance for a group of voxels was defined as the median of its performance over the voxels in the group ($\tilde{r}$). The data of all subjects were concatenated prior to analyzing the performance of the models.

In order to make sure that the differences in the performance of a model in different areas are not caused by the differences in the signal-to-noise ratios of the areas, the performance of the model in an area was corrected for the median of the noise ceilings of the voxels in the area (${\tilde{r}}^{*}$) (Kay et al., 2013). Briefly, we performed Monte Carlo simulations in which the correlation coefficient between a signal and a noisy signal is estimated. In each simulation, both the signal and the noise were drawn from a Gaussian distribution. The noisy signal was taken to be the summation of the signal sample and the noise sample. The parameters of the signal and the noise distributions were estimated from the 10 repeated measurements of the responses to the same stimuli. The noise distribution was assumed to be zero mean, and its variance was taken to be the variance of the standard errors of the data. The mean and the variance of the signal distribution were given as the mean of the data, and the difference between the variance of the data and the noise distribution, respectively. The medians of the correlation coefficients that were estimated in the simulations were taken to be the noise ceilings of the voxels, indicating the maximum performance that can be expected from the perfect model due to the noise in the data.

Permutation tests were used for comparing the performance of a model against chance level. First, data were randomly permuted over time for 200 times. Then, a separate model was trained and tested for each of the 200 permutations. Finally, the *p*-value was taken to be the fraction of the 200 permutations whose performance was greater than the actual performance. The performance was considered significant at α = 0.05 if the *p*-value was less than 0.05 (Bonferroni corrected for number of areas).

Bootstrapping was used for comparing the performance of two models over voxels in a ROI (i.e., all voxels or voxels in an area). For 10,000 repetitions, bootstrap samples (i.e., voxels) were drawn from the ROI with replacement, and the performance difference between the models over these voxels were estimated. The performance difference was considered significant at α = 0.05 if the 95% confidence interval of the sampled statistic did not cover zero (Bonferroni corrected for number of models).

## 3. Results

### 3.1. Comparison of response models

We evaluated the response models by comparing the performance of the response models in the (recurrent) RNN family and (feed-forward) ridge regression family in combination with the (high-level) W2V model and the (low-level) GIST model. Using two feature models of different levels ruled out any potential biases in the performance difference of the response models that can be caused by the feature models. Recall that the models in the RNN family (*G/L-10*/*50*/*100* models) differed in the type and number of artificial neurons, whereas the models in the ridge regression family (*R-C*/*R-CTD*/*R-F* models) differed in how they account for the hemodynamic delay.

Once the best response models among the RNN family and the ridge regression family were identified, we first compared their performance in detail. Particular attention was paid to the voxels where the performance of the models differed by more than an arbitrary threshold of *r* = 0.1. We then compared the performance of the best response model among the RNN family over the areas along the visual pathway.

#### 3.1.1. Comparison of the response models in combination with the semantic model

**Figure 2:**
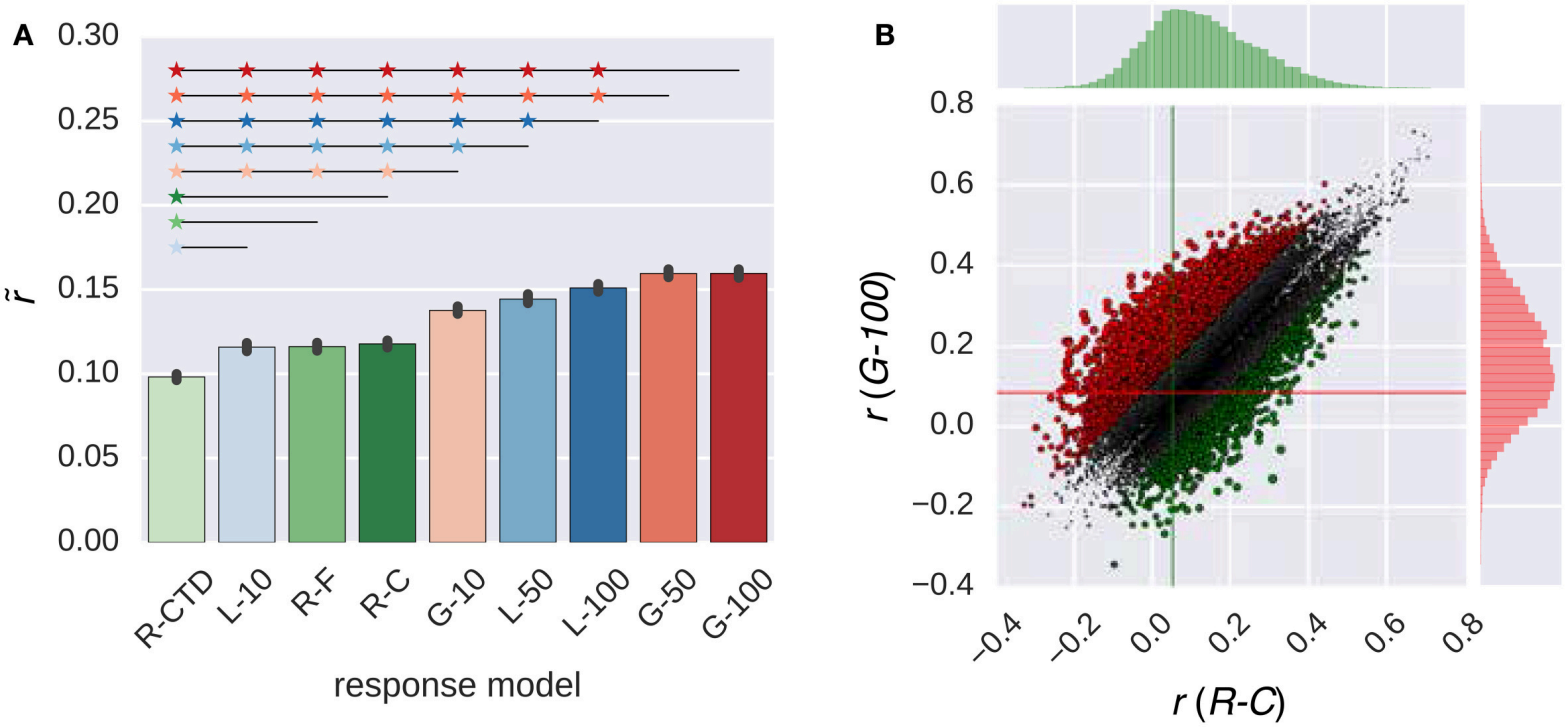
**Comparison of the response models in combination with the W2V model**. **(A)** Median performance of response models in RNN (*G-X* and *L-X*) and ridge regression (*R-X*) families over all voxels. Error bars indicate 95% confidence intervals (bootstrapping). Asterisks indicate significant performance difference. All of the individual bars depict significantly above chance-level performance (*p* < 0.05, permutation test). **(B)** Performance of best response models in RNN (*G-100* model) and ridge regression (*R-C* model) families over individual voxels. Points indicate voxels. Gray points indicate voxels where the performance difference is less than *r* = 0.1. Lines indicate (median) performance over all voxels.

Figure 2 compares the performance of all response models in combination with the W2V model. The performance of the models in the RNN family that had 50 or 100 artificial neurons was always significantly higher than that of all models in the ridge regression family (*p* ≤ 0.05, bootstrapping). However, the performance of the models in the same family was not always significantly different from each other. The performance of the *G-100* model was the highest among the RNN family ($\tilde{r}=0.16$), and that of the *R-C* model was the highest among the ridge regression family ($\tilde{r}\;=\;0.12$).

The performance of the *G-100* model and the *R-C* model differed from each other by more than the chosen threshold of *r* = 0.1 in 30% of the voxels. The performance of the *G-100* model was higher in 78% of these voxels ($\Delta \tilde{r}=0.17$), and that of the *R-C* model was higher in 22% of these voxels ($\Delta \tilde{r}=0.14$).

**Figure 3:**
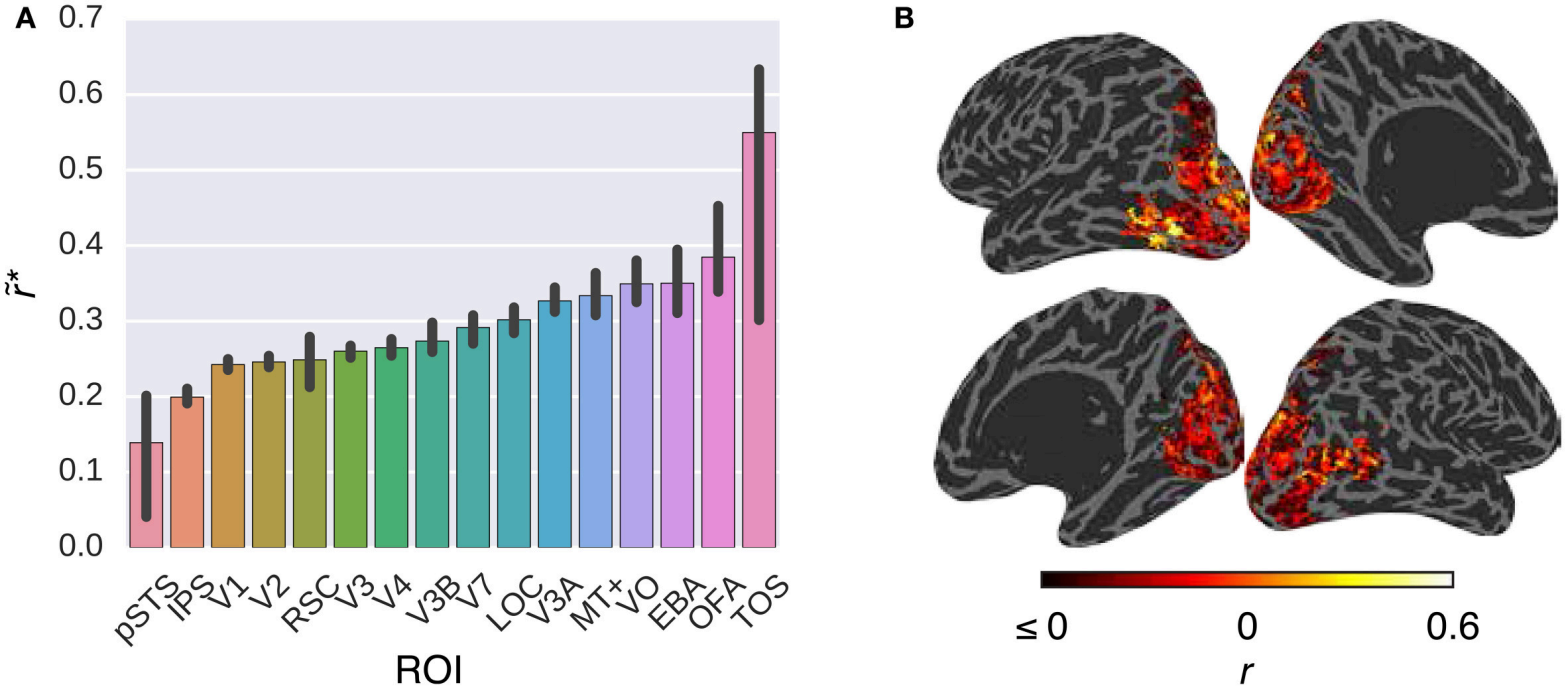
**Comparison of the *G-100* model in combination with the W2V model in different areas**. **(A)** Median noise ceiling controlled performance over all voxels in different areas. Error bars indicate 95% confidence intervals (bootstrapping). All of the individual bars depict significantly above chance-level performance (*p* < 0.05, permutation test). **(B)** Projection of performance to cortical surfaces of S3.

Figure 3 compares the performance of the *G-100* model in combination with the W2V model over the areas along the visual stream. While the performance of the model was significantly higher than chance throughout the areas (*p* ≤ 0.05, permutation test), it was particularly high in downstream areas. For example, it was the highest in TOS (${\tilde{r}}^{*}=0.55$), OFA (${\tilde{r}}^{*}=0.38$) and EBA (${\tilde{r}}^{*}=0.35$), and the lowest in pSTS (${\tilde{r}}^{*}=0.14$), IPS (${\tilde{r}}^{*}=0.20$) and V1 (${\tilde{r}}^{*}=0.24$).

#### 3.1.2. Comparison of the response models in combination with the low-level feature model

**Figure 4:**
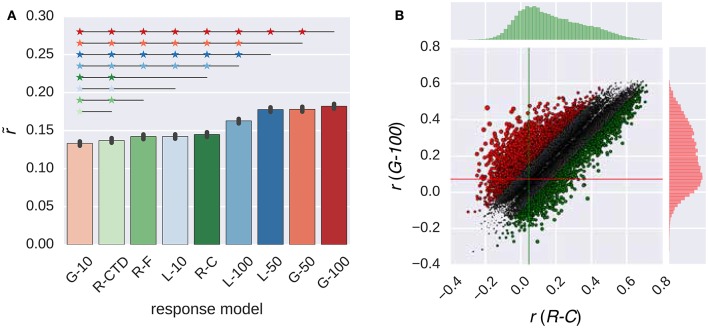
**Comparison of the response models in combination with the GIST model**. **(A)** Median performance of response models in RNN (*G-X* and *L-X*) and ridge regression (*R-X*) families over all voxels. Error bars indicate 95% confidence intervals (bootstrapping). Asterisks indicate significant performance difference. All of the individual bars depict significantly above chance-level performance (*p* < 0.05, permutation test). **(B)** Performance of best response models in RNN (*G-100* model) and ridge regression (*R-C model*) families over individual voxels. Points indicate voxels. Gray points indicate voxels where the performance difference is less than *r* = 0.1. Lines indicate median performance over all voxels.

Figure 4 compares the performance of the all response models in combination with the GIST model. The trends that were observed in this figure were similar to those that were observed in Figure 2. The *G-100* model was the best among the RNN family ($\tilde{r}=0.18$), and the *R-C* model was the best among the ridge regression family ($\tilde{r}=0.14$).

The *G-100* model and the *R-C* differed from each other by more than the threshold of *r* = 0.1 in 27% of the voxels. The *G-100* model was better in 66% of these voxels ($\Delta \tilde{r}=0.17$). The *R-C* model was better in 34% of these voxels ($\Delta \tilde{r}=0.14$).

**Figure 5:**
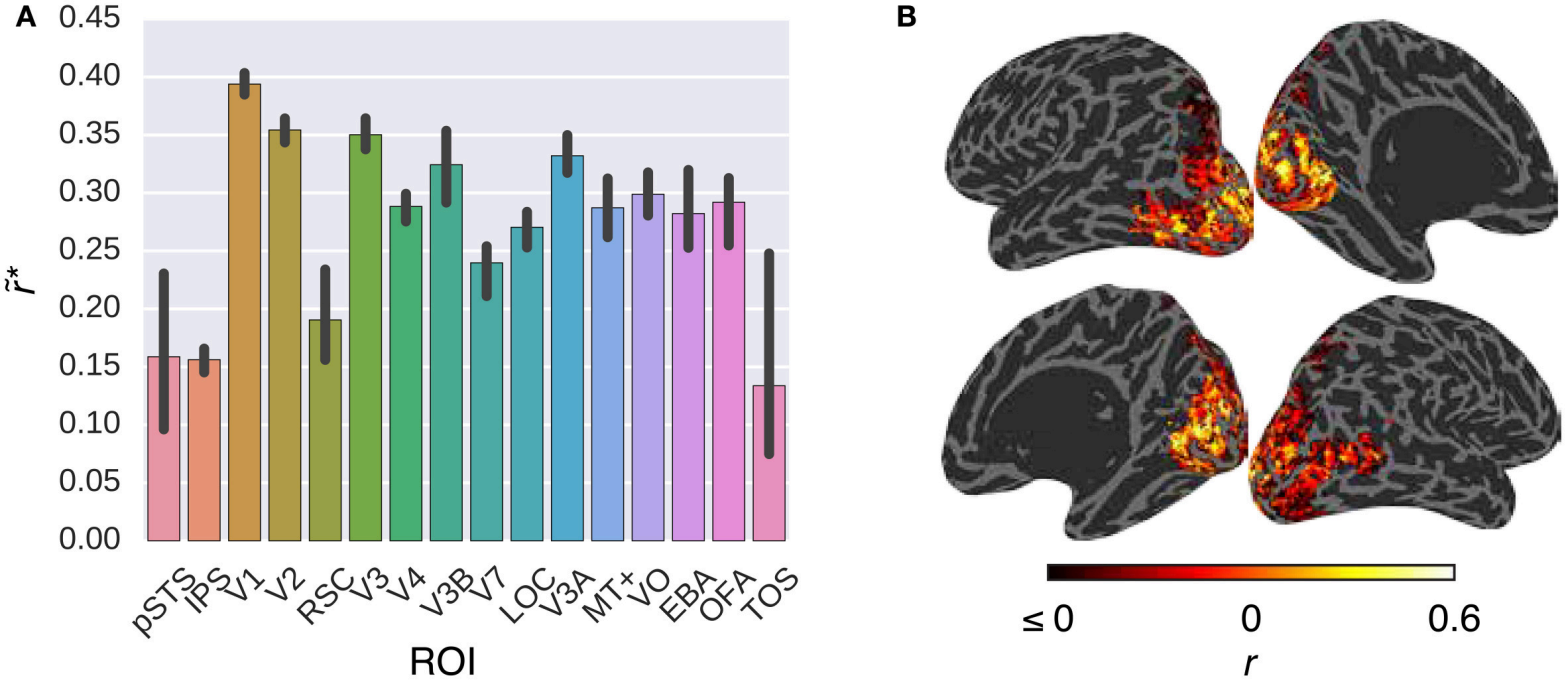
**Comparison of the *G-100* model in combination with the GIST model in different areas**. **(A)** Median noise ceiling controlled performance over all voxels in different areas. Error bars indicate 95% confidence intervals (bootstrapping). All of the individual bars depict significantly above chance-level performance (*p* < 0.05, permutation test). **(B)** Projection of performance to cortical surfaces of S3.

Figure 5 compares the performance of the *G-100* model in combination with the GIST model over the areas along the visual pathway. While the *G-100* model performed significantly better than chance throughout the areas (*p* ≤ 0.05, permutation test), it performed particularly well in upstream visual areas. For example, it performed the best in V1 (${\tilde{r}}^{*}=0.39$), V2 (${\tilde{r}}^{*}=0.35$) and V3 (${\tilde{r}}^{*}=0.35$), and the worst in TOS (${\tilde{r}}^{*}=0.13$), IPS (${\tilde{r}}^{*}=0.16$) and pSTS (${\tilde{r}}^{*}=0.16$).

### 3.2. Comparison of feature models

**Figure 6:**
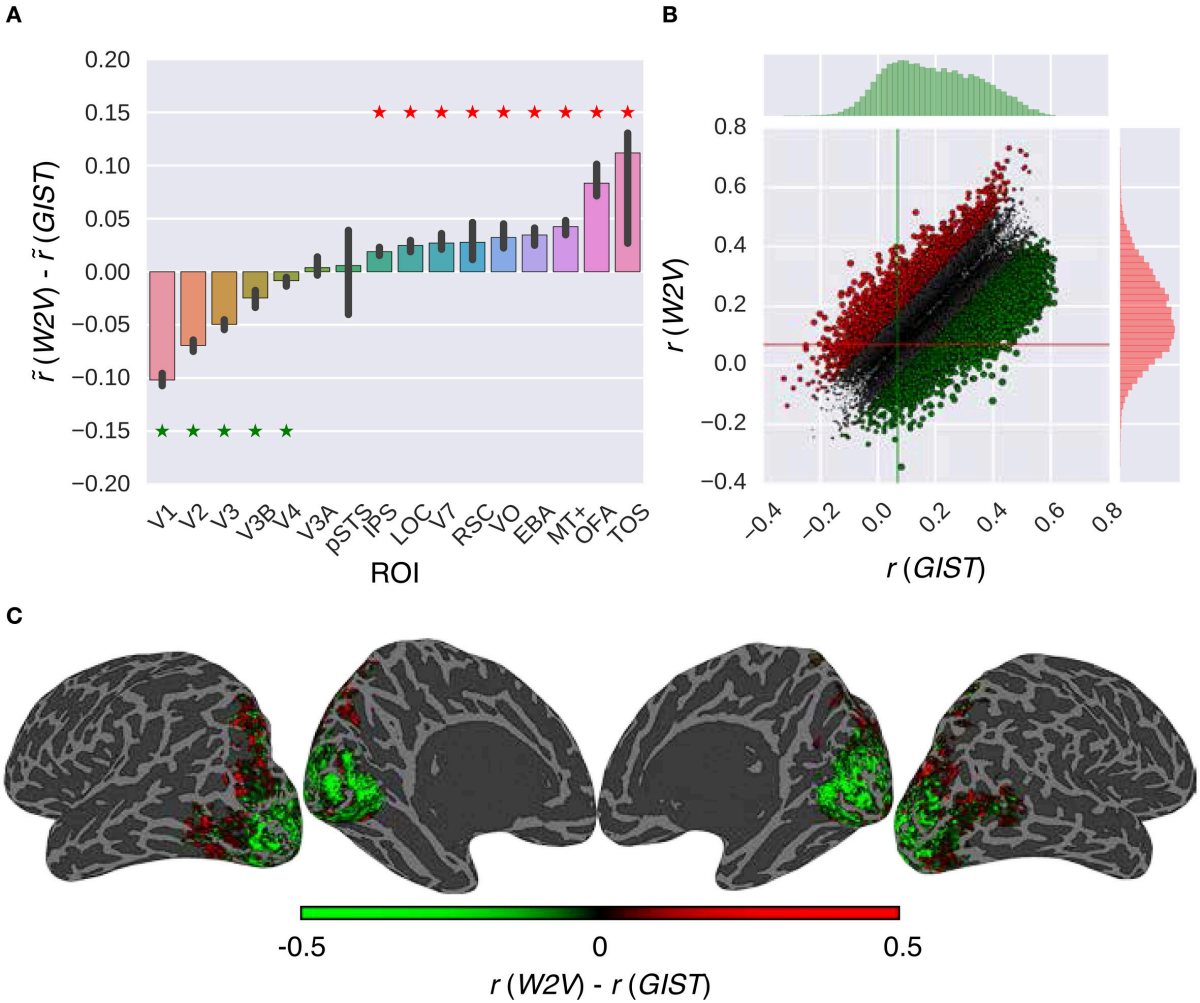
**Comparison of the feature models in combination with the *G-100* model**. **(A)** Median performance difference over all voxels in different areas. Asterisks indicate significant performance difference. Error bars indicate 95% confidence intervals (bootstrapping). **(B)** Performance over individual voxels. Points indicate voxels. Gray points indicate voxels where performance difference is less than *r* = 0.1. Lines indicate median performance over all voxels. **(C)** Projection of performance difference to cortical surfaces of S3.

Once the efficacy of the proposed RNN models was positively assessed, we performed a validation experiment in which we assessed the extent to which these models can replicate the earlier findings on the low-level and high-level subdivision of the visual cortex. This was accomplished by identifying the voxels that prefer semantic representations vs. low-level representations. Concretely, we compared the performance of the W2V model and the GIST model in combination with the *G-100* model (Figure 6).

The performance of the models was significantly different in all areas along the visual stream except for pSTS and V3A (*p* ≤ 0.05, bootstrapping). This difference was in favor of semantic representations in downstream areas and low-level representations in upstream areas. The largest difference in favor of semantic representations was in TOS ($\Delta \tilde{r}=0.11$), OFA ($\Delta \tilde{r}\;=\;0.08$) and MT+ ($\Delta \tilde{r}=0.04$), and low-level representations was in V1 ($\Delta \tilde{r}=0.10$), V2 ($\Delta \tilde{r}=0.07$) and V3 ($\Delta \tilde{r}=0.05$).

Thirty-nine percent of the voxels preferred either representation by more than the arbitrary threshold of *r* = 0.1. Thirty-four percent of these voxels preferred semantic representations ($\Delta \tilde{r}=0.16$), and 66% percent of these voxels preferred low-level representations ($\Delta \tilde{r}=0.18$).

These results are in line with a large number of earlier work that showed similar dissociations between the representations of the upstream and downstream visual areas (Mishkin et al., 1983; Naselaris et al., 2009; DiCarlo et al., 2012; Güçlü and van Gerven, 2015a).

### 3.3. Analysis of internal representations

**Figure 7:**
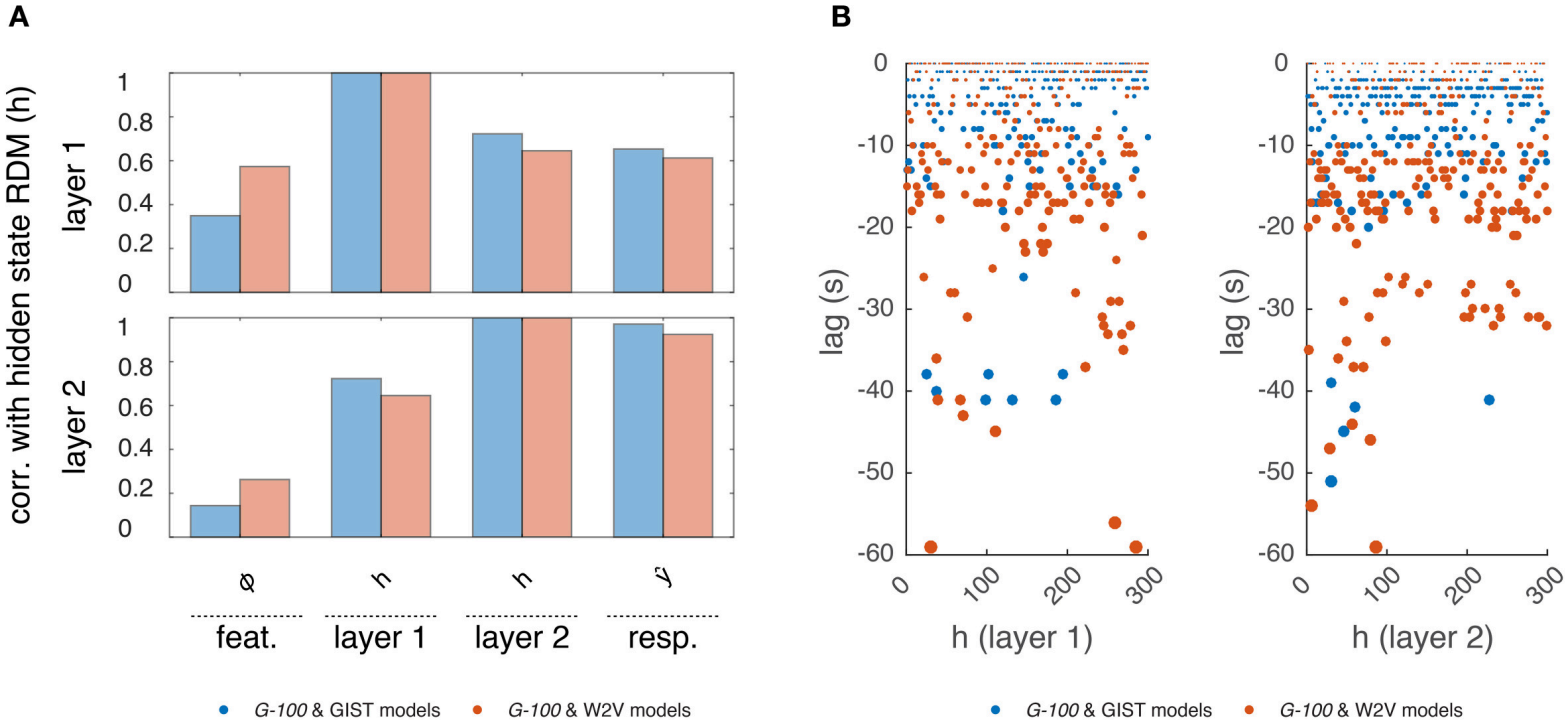
**Internal representations of the *G-100* model**. **(A)** Correlation between representational dissimilarity matrices of layer 1 and layer 2 hidden states with each other as well as with those of features and predicted responses. **(B)** Temporal selectivity of layer 1 and layer 2 hidden units. Points indicate lags at which cross-correlations between hidden states and features are highest.

Next, to gain insight into the temporal dependencies captured by the *G-100* model, we analyzed its internal representations (Figure 7).

First, we investigated how the hidden states of the RNN depend on its inputs and output. We constructed representational dissimilarity matrices (RDMs) of the stimulus sequence in the test set at different stages of the processing pipeline and averaged them over subjects (Kriegeskorte et al., 2008). Per feature model, this resulted in one RDM for the features, two RDMs for the layer 1 and layer 2 hidden states and one RDM for the predicted responses. We correlated the upper triangular parts of the RDMs with one another, which resulted in a value indicating how much the hidden states of the RNN were modulated by its inputs and how much they modulated its outputs at a given time point. We found a gradual increase in correlations of the RDMs. That is, the RDMs at each stage were more correlated with those at the next stage compared to those at the previous stages. Importantly, the hidden state RDMs were highly correlated with the predicted response RDMs (*r* = 0.61 and *r* = 0.93 for layers 1 and 2, respectively) but less so with the feature RDMs (*r* = 0.39 and *r* = 0.21 for layers 1 and 2, respectively). This means that while the hidden states of the RNN modulated its outputs at a given time point, they were not modulated by its inputs to the same extent at the same time point. This suggests that a substantial part of the output at a given time-point is not directly related to the input at the same time-point, but instead to previous time-points. That is, the RNN learned to use the input history to make its predictions as expected.

Then, we investigated which time points in the input history were used by the RNN to make its predictions. We cross-correlated each hidden state with each stimulus feature, and averaged the cross-correlations over the features, which resulted in a value indicating how much a hidden state is selective to different time points in the input history. The time point at which this value was at its maximum was taken as the optimal lag of that hidden unit. We found that different hidden units had different optimal lags. The majority of the hidden units had optimal lags up to -20 s, which are likely capturing the hemodynamic factors. However, there was a non-negligible number of hidden units with optimal lags beyond this period, which might be capturing other cognitive/neuronal factors or factors related to stimulus/feature statistics. It should be noted that not all hidden units, in particular those with extensive lags, can be attributed to any of these factors, and their behavior might be induced by model definition or estimation. Furthermore, the optimal lags of the hidden units in the *W2V* based model were on average significantly higher than those in the *GIST* based model (μ = −9.6 s vs. μ = −4.9 s, *p* < 0.05, two-sample *t*-test), which might reflect the differences in the statistics of the features that the models are based on. That is, high-level semantic features tend to be more persistent than the low-level structural features across the input sequence. For example, over a given video sequence, distribution of objects in a scene change relatively slowly compared to that of the edges in the scene.

### 3.4. Estimation of voxel-specific HRFs

Traditionally, models have used analytically derived (Friston et al., 1998) or statistically estimated (Dale, 1999; Glover, 1999) HRFs such as the linear models considered here. Estimation of voxel-specific HRFs is an important problem since using the same HRF for all voxels ignores the variability of the hemodynamic response across the brain, which might adversely affect the model performance. Recent developments have focused on the derivation and estimation of more accurate HRFs. For example, Aquino et al. (2014) has shown that HRFs can be analytically derived from physiology, and Pedregosa et al. (2015) has shown that HRFs can be efficiently estimated from data. Note that, while the methods for statistically estimating HRFs are particularly suited for use in block designs and event related designs, they are less straightforward to use in continuous designs such as the one considered here.

**Figure 8:**
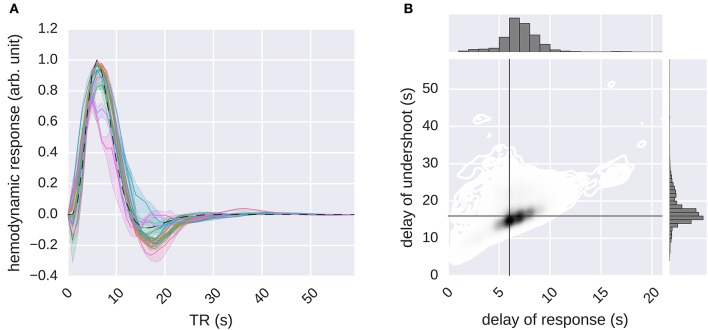
**Estimation of the hemodynamic response functions**. The *G-100* model was stimulated with an impulse. The impulse response was processed by normalizing its baseline and scale. The result was taken as the HRF. **(A)** Median hemodynamic response functions of all voxels in different areas. Error bands indicate 68% confidence intervals (bootstrapping). Different colors indicate different areas. Dashed line indicates canonical hemodynamic response function. **(B)** Delays of responses and undershoots of all voxels. Black lines indicate canonical delays.

As demonstrated in the previous subsection, one important advantage of the response models in the RNN family is that they can capture certain temporal dependencies in the data, which might correspond to the HRFs of voxels. Here, we evaluate the voxel-specific HRFs that are obtained by stimulating the *G-100* model with an impulse. We used both feature models in combination with the *G-100* model to estimate the HRFs of the voxels where the performance of any model combination was significantly higher than chance (51% of the voxels, *p* ≤ 0.05, Student's *t*-test, Bonferroni correction) (Figure 8). The W2V and *G-100* models were used to estimate the HRFs of the voxels where their performance was higher than that of the GIST and *G-100* models, and vice versa.

It was found that the global shape of the estimated HRFs was similar to that of the canonical HRF. However, there was a considerable spread in the estimated delays of responses and the delays of undershoots (median delay of response = 6.57 ± 0.02 s, median delay of undershoot = 16.95 ± 0.04 s), with the delays of responses being significantly correlated with the delays of undershoots (Pearson's *r* = 0.45, *p* ≤ 0.05, Student's *t*-test).

These results demonstrate that RNNs can not only learn (stimulus) feature-response relationships but also can estimate HRFs of voxels, which in turn demonstrate that the nonlinear temporal dynamics that are learned by the RNNs capture biologically relevant temporal dependencies. Furthermore, the variability in the estimated voxel-specific HRFs revealed by the recurrent models might provide a partial explanation of the performance difference between the recurrent and ridge regression models since the ridge regression models use fixed or restricted HRFs, making it difficult for them to take such variability into account.

## 4. Discussion

Understanding how the human brain responds to its environment is a key objective in neuroscience. This study has shown that recurrent neural networks are exquisitely capable of capturing how brain responses are induced by sensory stimulation, outperforming established approaches augmented with ridge regression. This increased sensitivity has important consequences for future studies in this area.

### 4.1. Testing hypotheses about brain function

Like any other encoding model, RNN based encoding models can be used to test hypotheses about neural representations (Naselaris et al., 2011). That is, they can be used to test whether a particular feature model outperforms alternative feature models when it comes to explaining observed data. As such, we have shown that a low-level visual feature model explains responses in upstream visual areas well, whereas a high-level semantic model explains responses in downstream visual areas well, conforming to the well established early and high-level subdivision of the visual cortex (Mishkin et al., 1983; Naselaris et al., 2009; DiCarlo et al., 2012; Güçlü and van Gerven, 2015a).

Furthermore, RNN-based encoding models can also be used to test hypotheses about the temporal dependencies between features and responses. For example, by constraining the temporal memory capacities of the RNN units, one can identify the optimal scale of the temporal dependencies that different brain regions are selective to.

Here, we used RNNs as response models in an encoding framework. That is, they were used to predict responses to features that were extracted from stimuli with separate feature models. However, use cases of RNNs are not limited to this setting. For example, RNN models can be used as feature models instead of response models in the encoding framework. Like CNNs, RNNs are being used to solve various problems in fields ranging from computer vision (Gregor et al., 2015) to computational linguistics (Zaremba et al., 2014). Internal representations of task-optimized CNNs were shown to correspond to neural representations in different brain regions (Kriegeskorte, 2015; Yamins and DiCarlo, 2016b). It would be interesting to see if the internal representations of task-based RNNs have similar correlates in the brain. For example, it was recently shown that RNNs develop representations that are reminiscent of their biological counterparts when they learn to solve a spatial navigation task (Kanitscheider and Fiete, 2016). Such representations may turn out to be predictive of brain responses recorded during similar tasks.

### 4.2. Limitations of RNNs for investigating neural representations

RNNs can process arbitrary input sequences in theory. However, they have an important limitation in practice. Like any other contemporary neural network architecture, typical RNN architectures have a very large number of free parameters. Therefore, a very large amount of training data is required for accurately estimating RNN models without overfitting. While there are several methods to combat overfitting in RNNs like different variants of dropout (Hinton et al., 2012; Zaremba et al., 2014; Semeniuta et al., 2016), it is still an important issue to which particular attention needs to be paid.

This can also be the reason why gated recurrent unit architectures were shown to outperform LSTM architectures. That is, the performance difference between the two types of architectures is likely to be caused by difficulties in model estimation in the current data regime rather than one architecture being better suited to the problem at hand than the other.

This also means that RNN models will face difficulties when trying to predict responses to very high-dimensional stimulus features such as the internal representations of convolutional neural networks which range from thousands to hundreds of thousands dimensions. For such features, dimensionality reduction techniques can be utilized for reducing the feature dimensionality to a range that can be handled with RNNs in scenarios with either insufficient computational resources or training data.

Linear response models have been used with great success in the past for gaining insights into neural representations. They have been particularly useful since linear mappings make it easy to interpret factors driving response predictions. One might argue that the nonlinearities introduced by RNNs make the interpretation harder compared to linear mappings. However, the relative difficulty of interpretation is a direct consequence of more accurate response predictions, which can be beneficial in certain scenarios. For example, it was shown that systematic nonlinearities that are not taken into account by linear mappings can lead to less accurate response predictions and tuning functions of V1 voxels (Vu et al., 2011). Furthermore, since more accurate response predictions lead to higher statistical power, the improved model fit afforded by RNNs might make detection of more subtle effects possible. Moreover, when the goal is to compare different feature models, such as the GIST and W2V models used here, maximizing explained variance might become the main criterion of interest. That is, linear models might lead to misleading performance differences between the encoding models in the cases where their assumptions about the underlying temporal dynamics do not hold. In such cases, it would be particularly important to fit the response models as accurately as possible as to ensure that the observed performance difference between two encoding models is driven by their underlying feature representations and not suboptimal model fits. Therefore, RNNs will be particularly useful in settings where temporal dynamics are of primary interest. Finally, combining the present work with recent developments on understanding RNN representations (Karpathy et al., 2015) is expected to improve the interpretations of factors driving response predictions.

### 4.3. Capturing temporal dependencies

RNNs can use their internal memories to capture the temporal dependencies in data. In the context of modeling the dynamics of brain activity in response to naturalistic stimuli, these dependencies can be caused by factors such as neurovascular coupling or stimulus-induced cognitive processes. By providing an RNN with an impulse on the input side, it was shown that, effectively, the RNN learns to represent voxel-specific hemodynamic responses. Importantly, the RNNs allowed us to estimate these HRFs from data collected under a continuous design. To the best of our knowledge this is the first time it has been shown that this is possible in practice. By analyzing the internal representations of an RNN, it was also shown that the RNN learns to represent information from stimulus features at past time points beyond the range of neurovascular coupling. Hence, the predictions of observed brain responses are likely induced by stimulus-related, cognitive or neural factors on top of the hemodynamic response.

### 4.4. Isolating neural and hemodynamic components

In the introduction, we motivated the use of RNNs as a generic parameterization of any non-linear convolution of stimulus features to hemodynamic responses. Crucially, this could cover both neuronal and hemodynamic convolution. In other words, our black box approach allows for a neuronal convolution of stimulus feature input to produce a neuronal response that is subsequently convolved by hemodynamic operators to produce the observed outcome. This facility may explain the increased cross-validation accuracy observed in our analyses (over and above more restricted models of hemodynamic convolution). In other words, the procedure detailed in this paper can accommodate neuronal convolutions that may be precluded in conventional models.

The cost of this flexibility is that we cannot separate the neuronal and hemodynamic components of the convolution. This follows from the fact that the RNN parameterization does not make an explicit distinction between neuronal and hemodynamic processes. To properly understand the relative contribution of these formally distinct processes, one would have to use a generative model approach with biologically plausible prior constraints on the neuronal and hemodynamic parts of the convolution. This is precisely the objective of dynamic causal modeling that equips a system of neuronal dynamics (and implicit recurrent connectivity) with a hemodynamic model based upon known biophysics (Friston et al., 2003). It would therefore be interesting to examine the form of RNNs in relation to existing dynamic causal models that have a similar architecture.

### 4.5. Conclusions

We have shown for the first time that RNNs can be used to predict how the human brain processes sensory information. Whereas classical connectionist research has focused on the use of RNNs as models of cognitive processing (Elman, 1993), the present work has shown that RNNs can also be used to probe the hemodynamic correlates of ongoing cognitive processes induced by dynamically changing naturalistic sensory stimuli. The ability of RNNs to learn about long-range temporal dependencies provides the flexibility to couple ongoing sensory stimuli that induce various cognitive processes with delayed measurements of brain activity that depend on such processes. This end-to-end training approach can be applied to any neuroscientific experiment in which sensory inputs are coupled to observed neural responses.

### 4.6. Data sharing

The data set that was used in this paper was originally published in Nishimoto et al. (2011) and is available at Nishimoto et al. (2014). The code that was used in this paper is provided at http://www.ccnlab.net/.

## Ethics statement

Human fMRI data set that was used in this study was taken from the public data sharing repository http://crcns.org/. The original study was approved by the local ethics committee (Committee for the Protection of Human Subjects at University of California, Berkeley).

## Author contributions

UG and MvG designed research; UG performed research; UG and MvG contributed unpublished reagents/analytic tools; UG analyzed data; UG and MvG wrote the paper.

## Funding

This research was supported by VIDI grant number 639.072.513 of the Netherlands Organization for Scientific Research (NWO).

### Conflict of interest statement

The authors declare that the research was conducted in the absence of any commercial or financial relationships that could be construed as a potential conflict of interest.

## Footnotes

^1^The cross-validated correlation coefficient automatically penalizes for model complexity and therefore can be used as a proxy for model evidence.

## References

[B1] Agrawal P. Stansbury D. Malik J. Gallant J. L. (2014). Pixels to voxels: modeling visual representation in the human brain. arXiv:1407.5104 [q-bio.NC].

[B2] Aquino K. M. Robinson P. A. Drysdale P. M. (2014). Spatiotemporal hemodynamic response functions derived from physiology. J. Theor. Biol. 347, 118–136. 10.1016/j.jtbi.2013.12.027 24398024

[B3] Cadieu C. F. Hong H. Yamins D. L. Pinto N. Ardila D. Solomon E. A.. (2014). Deep neural networks rival the representation of primate IT cortex for core visual object recognition. PLoS Comput. Biol. 10:e1003963. 10.1371/journal.pcbi.1003963 25521294 PMC4270441

[B4] Cho K. van Merrienboer B. Gulcehre C. Bahdanau D. Bougares F. Schwenk H. (2014). Learning phrase representations using RNN encoder-decoder for statistical machine translation. arXiv:1406.1078 [cs.CL].

[B5] Cichy R. M. Khosla A. Pantazis D. Torralba A. Oliva A. (2016). Deep neural networks predict hierarchical spatio-temporal cortical dynamics of human visual object recognition. arXiv:1601.02970 [cs.CV]. 10.1038/srep27755 PMC4901271 27282108

[B6] Dale A. M. (1999). Optimal experimental design for event-related fMRI. Hum. Brain Mapp. 8, 109–114. 10524601 10.1002/(SICI)1097-0193(1999)8:2/3<109::AID-HBM7>3.0.CO;2-W PMC6873302

[B7] DiCarlo J. J. Zoccolan D. Rust N. C. (2012). How does the brain solve visual object recognition? Neuron 73, 415–434. 10.1016/j.neuron.2012.01.010 22325196 PMC3306444

[B8] Eickenberg M. Gramfort A. Varoquaux G. Thirion B. (2016). Seeing it all: convolutional network layers map the function of the human visual system. NeuroImage. 10.1016/j.neuroimage.2016.10.001 [Epub ahead of print]. 27777172

[B9] Elman J. L. (1993). Learning and development in neural networks - the importance of prior experience. Cognition 48, 71–99. 10.1016/0010-0277(93)90058-4 8403835

[B10] Felsen G. Dan Y. (2005). A natural approach to studying vision. Nat. Neurosci. 8, 1643–1646. 10.1038/nn1608 16306891

[B11] Friston K. J. Harrison L. Penny W. (2003). Dynamic causal modelling. Neuroimage 19, 1273–1302. 10.1016/S1053-8119(03)00202-7 12948688

[B12] Friston K. J. Holmes A. P. Worsley K. J. Poline J.-P. Frith C. D. Frackowiak R. S. J. (1994). Statistical parametric maps in functional imaging: a general linear approach. Hum. Brain Mapp. 2, 189–210. 10.1002/hbm.460020402

[B13] Friston K. J. Josephs O. Rees G. Turner R. (1998). Nonlinear event-related responses in fMRI. Magn. Reson. Med. 39, 41–52. 10.1002/mrm.1910390109 9438436

[B14] Friston K. J. Mechelli A. Turner R. Price C. J. (2000). Nonlinear responses in fMRI: the Balloon model, Volterra kernels, and other hemodynamics. Neuroimage 12, 466–477. 10.1006/nimg.2000.0630 10988040

[B15] Fyshe A. Talukdar P. Murphy B. Mitchell T. (2013). Documents and dependencies: an exploration of vector space models for semantic composition, in Documents and dependencies: an exploration of vector space models for semantic composition, (Sofia).

[B16] Glover G. H. (1999). Deconvolution of impulse response in event-related BOLD fMRI. NeuroImage 9, 416–429. 10.1006/nimg.1998.0419 10191170

[B17] Graves A. (2013). Generating sequences with recurrent neural networks. arXiv:1308.0850 [cs.NE].

[B18] Graves A. Liwicki M. Fernández S. Bertolami R. Bunke H. Schmidhuber J. (2009). A novel connectionist system for unconstrained handwriting recognition. IEEE Trans. Patt. Anal. Mach. Intell. 31, 855–868. 10.1109/TPAMI.2008.137 19299860

[B19] Gregor K. Danihelka I. Graves A. Jimenez Rezende D. Wierstra D. (2015). DRAW: A recurrent neural network for image generation. arXiv:1502.04623 [cs.CV].

[B20] Groen I. I. Ghebreab S. Prins H. Lamme V. A. Scholte H. S. (2013). From image statistics to scene gist: evoked neural activity reveals transition from low-level natural image structure to scene category. J. Neurosci. 33, 18814–18824. 10.1523/JNEUROSCI.3128-13.2013 24285888 PMC6618700

[B21] Güçlü U. van Gerven M. A. (2014). Unsupervised feature learning improves prediction of human brain activity in response to natural images. PLoS Comput. Biol. 10:e1003724. 10.1371/journal.pcbi.1003724 25101625 PMC4125038

[B22] Güçlü U. van Gerven M. A. J. (2015a). Deep neural networks reveal a gradient in the complexity of neural representations across the ventral stream. J. Neurosci. 35, 10005–10014. 10.1523/JNEUROSCI.5023-14.2015 26157000 PMC6605414

[B23] Güçlü U. van Gerven M. A. J. (2015b). Increasingly complex representations of natural movies across the dorsal stream are shared between subjects. NeuroImage. 145, 329–336. 10.1016/j.neuroimage.2015.12.036 26724778

[B24] Güçlü U. van Gerven M. A. J. (2015c). Semantic vector space models predict neural responses to complex visual stimuli. arXiv:1510.04738 [q-bio.NC].

[B25] Hansen K. A. David S. V. Gallant J. L. (2004). Parametric reverse correlation reveals spatial linearity of retinotopic human V1 BOLD response. NeuroImage 23, 233–241. 10.1016/j.neuroimage.2004.05.012 15325370

[B26] Hinton G. E. Srivastava N. Krizhevsky A. Sutskever I. Salakhutdinov R. R. (2012). Improving neural networks by preventing co-adaptation of feature detectors. arXiv:1207.0580 [cs.NE].

[B27] Hochreiter S. Schmidhuber J. (1997). Long short-term memory. Neural Comput. 9, 1735–1780. 10.1162/neco.1997.9.8.1735 9377276

[B28] Huth A. G. Nishimoto S. Vu A. T. Gallant J. L. (2012). A continuous semantic space describes the representation of thousands of object and action categories across the human brain. Neuron 76, 1210–1224. 10.1016/j.neuron.2012.10.014 23259955 PMC3556488

[B29] Kanitscheider I. Fiete I. (2016). Training recurrent networks to generate hypotheses about how the brain solves hard navigation problems. arXiv:1609.09059 [q-bio.nc].

[B30] Karpathy A. Johnson J. Fei-Fei L. (2015). Visualizing and understanding recurrent networks. arXiv:1506.02078 [cs.LG].

[B31] Kay K. N. Winawer J. Mezer A. Wandell B. A. (2013). Compressive spatial summation in human visual cortex. J. Neurophysiol. 110, 481–494. 10.1152/jn.00105.2013 23615546 PMC3727075

[B32] Khaligh-Razavi S.-M. Kriegeskorte N. (2014). Deep supervised, but not unsupervised, models may explain IT cortical representation. PLoS Comput. Biol. 10:e1003915. 10.1371/journal.pcbi.1003915 25375136 PMC4222664

[B33] Kingma D. Ba J. (2014). Adam: a method for stochastic optimization. arXiv:1412.6980 [cs.LG].

[B34] Kriegeskorte N. (2015). Deep neural networks: a new framework for modeling biological vision and brain information processing. Ann. Rev. Vis. Sci. 1, 417–446. 10.1146/annurev-vision-082114-035447 28532370

[B35] Kriegeskorte N. Mur M. Bandettini P. (2008). Representational similarity analysis - connecting the branches of systems neuroscience. Front. Syst. Neurosci. 2:4. 10.3389/neuro.06.004.2008 19104670 PMC2605405

[B36] Leeds D. D. Seibert D. A. Pyles J. A. Tarr M. J. (2013). Comparing visual representations across human fMRI and computational vision. J. Vis. 13, 25. 10.1167/13.13.25 24273227 PMC3839261

[B37] Logothetis N. K. Wandell B. A. (2004). Interpreting the BOLD signal. Ann. Rev. Physiol. 66, 735–769. 10.1146/annurev.physiol.66.082602.092845 14977420

[B38] Mikolov T. Chen K. Corrado G. Dean J. (2013a). Efficient estimation of word representations in vector space. arXiv:1301.3781 [cs.CL].

[B39] Mikolov T. Sutskever I. Chen K. Corrado G. Dean J. (2013b). Distributed representations of words and phrases and their compositionality. arXiv:1310.4546 [cs.CL].

[B40] Mikolov T. Yih W.-T. Zweig G. (2013c). Linguistic regularities in continuous space word representations, in Linguistic regularities in continuous space word representations, (Atlanta, GA).

[B41] Mishkin M. Ungerleider L. G. Macko K. A. (1983). Object vision and spatial vision: two cortical pathways. Trends Neurosci. 6, 414–417. 10.1016/0166-2236(83)90190-X

[B42] Mitchell T. M. Shinkareva S. V. Carlson A. Chang K. M. Malave V. L. Mason R. A.. (2008). Predicting human brain activity associated with the meanings of nouns. Science 320, 1191–1195. 10.1126/science.1152876 18511683

[B43] Murphy B. Talukdar P. Mitchell T. (2012). Selecting corpus-semantic models for neurolinguistic decoding, in Proceedings of First Joint Conference on Lexical and Computational Semantics (Montréal, QC).

[B44] Naselaris T. Kay K. N. Nishimoto S. Gallant J. L. (2011). Encoding and decoding in fMRI. NeuroImage 56, 400–410. 10.1016/j.neuroimage.2010.07.073 20691790 PMC3037423

[B45] Naselaris T. Prenger R. J. Kay K. N. Oliver M. Gallant J. L. (2009). Bayesian reconstruction of natural images from human brain activity. Neuron 63, 902–915. 10.1016/j.neuron.2009.09.006 19778517 PMC5553889

[B46] Nishida S. Huth A. Gallant J. L. Nishimoto S. (2015). Word statistics in large-scale texts explain the human cortical semantic representation of objects, actions, and impressions, in The 45th Annual Meeting of the Society for Neuroscience (Chicago, IL).

[B47] Nishimoto S. Vu A. T. Naselaris T. Benjamini Y. Yu B. Gallant J. L. (2011). Reconstructing visual experiences from brain activity evoked by natural movies. Curr. Biol. 21, 1641–1646. 10.1016/j.cub.2011.08.031 21945275 PMC3326357

[B48] Nishimoto S. Vu A. T. Naselaris T. Benjamini Y. Yu B. Gallant J. L (2014). Gallant Lab Natural Movie 4T fMRI Data. Available online at: http://CRCNS.org

[B49] Norris D. G. (2006). Principles of magnetic resonance assessment of brain function. J. Magn. Reson. Imaging 23, 794–807. 10.1002/jmri.20587 16649206

[B50] Oliva A. Torralba A. (2001). Modeling the shape of the scene: a holistic representation of the spatial envelope. Int. J. Comput. Vis. 42, 145–175. 10.1023/A:1011139631724

[B51] Pedregosa F. Eickenberg M. Ciuciu P. Thirion B. Gramfort A. (2015). Data-driven HRF estimation for encoding and decoding models. NeuroImage 104, 209–220. 10.1016/j.neuroimage.2014.09.060 25304775

[B52] Sak H. Senior A. Beaufays F. (2014). Long short-term memory based recurrent neural network architectures for large vocabulary speech recognition. arXiv:1402.1128 [cs.NE].

[B53] Semeniuta S. Severyn A. Barth E. (2016). Recurrent dropout without memory loss. arXiv:1603.05118 [cs.CL].

[B54] Sutskever I. Martens J. Hinton G. (2011). Generating text with recurrent neural networks, in Proceedings of the 28th International Conference on Machine Learning (Bellevue, WA).

[B55] Vu V. Q. Ravikumar P. Naselaris T. Kay K. N. Gallant J. L. Yu B. (2011). Encoding and decoding v1 fMRI responses to natural images with sparse nonparametric models. Ann. Appl. Stat. 5, 1159–1182. 10.1214/11-AOAS476 22523529 PMC3329873

[B56] Wray J. Green G. G. R. (1994). Calculation of the Volterra kernels of non-linear dynamic systems using an artificial neural network. Biol. Cybern. 71, 187–195. 10.1007/BF00202758

[B57] Yamins D. L. K. DiCarlo J. J. (2016a). Eight open questions in the computational modeling of higher sensory cortex. Curr. Opin. Neurobiol. 37, 114–120. 10.1016/j.conb.2016.02.001 26921828

[B58] Yamins D. L. K. DiCarlo J. J. (2016b). Using goal-driven deep learning models to understand sensory cortex. Nat. Neurosci. 19, 356–365. 10.1038/nn.4244 26906502

[B59] Yamins D. L. K. Hong H. Cadieu C. F. Solomon E. A. Seibert D. DiCarlo J. J. (2014). Performance-optimized hierarchical models predict neural responses in higher visual cortex. Proc. Natl. Acad. Sci. U.S.A. 111, 8619–8624. 10.1073/pnas.1403112111 24812127 PMC4060707

[B60] Zaremba W. Sutskever I. Vinyals O. (2014). Recurrent neural network regularization. arXiv:1409.2329 [cs.NE].

